# The 1990 Pittsburgh Conference: Scientific and commercial report

**DOI:** 10.1155/S1463924690000086

**Published:** 1990


Journal of Automatic Chemistry, Vol. 12, No. 2 (March–April 1990), pp. 60–79

### The 1990 Pittsburgh Conference: Scientific and commercial report

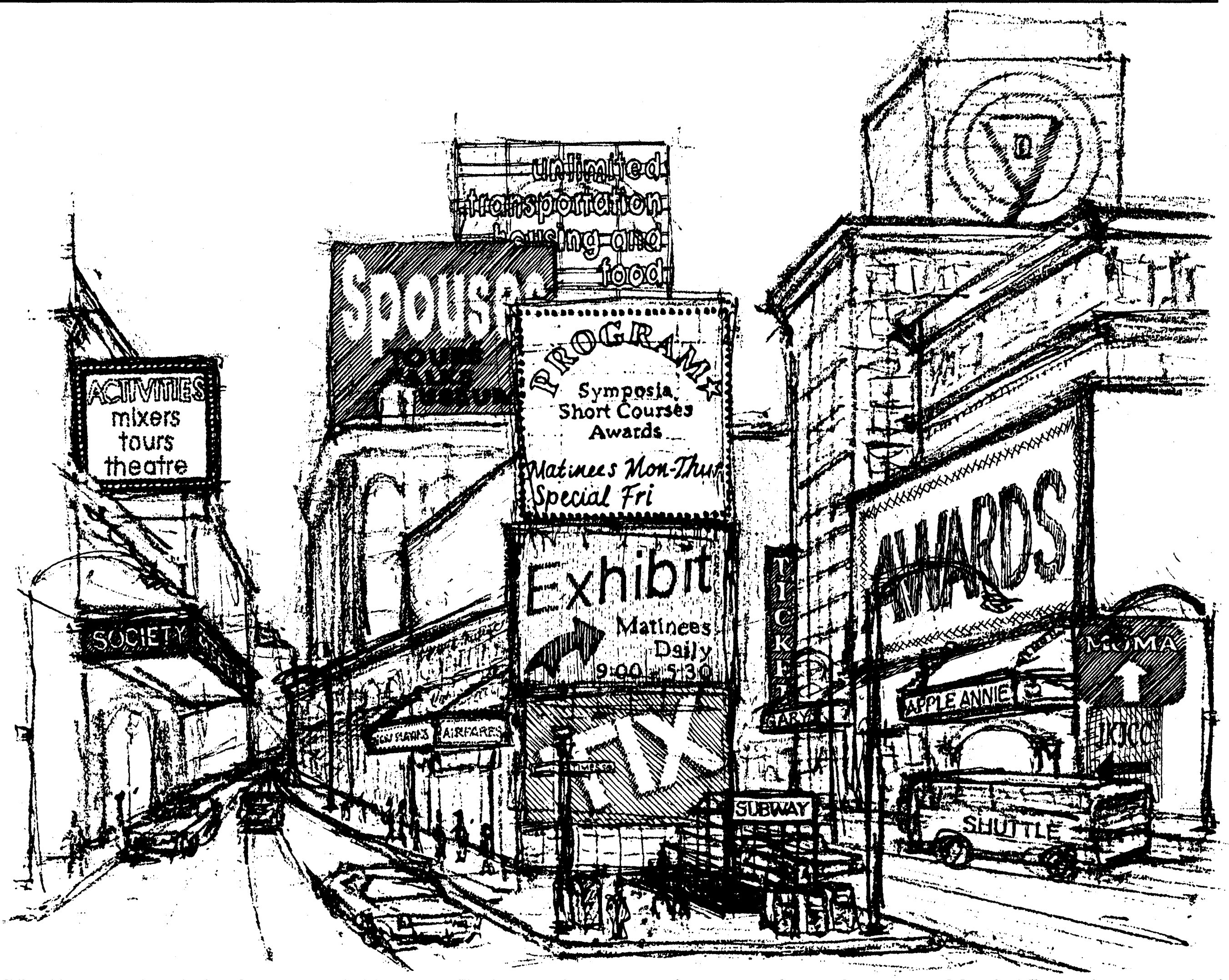

*The 1990 Pittsburgh Conference was held at New York's Jacob K. Javits Convention Center from 5 to 9 March. The conference involved around 1400 scientific presentations and registration on the first day was just short of 26 000. For the benefit of those users who were unable to go to the 1990 Pittsburgh Conference, and as a permanent record for those who did,* JAC *has decided to provide a special report containing the abstracts of the most relevant presentations and descriptions of new instruments.*

*The Editor wishes to thank the 1990 Program Chairman for kind permission to reproduce the abstracts.*

*The next Pittsburgh Conference will be held in Chicago from 4 to 8 March 1991. Details from The Pittsburgh Conference, 300 Penn Center Boulevard, Suite 332, Pittsburgh, Pennsylvania 15235, USA.*

### Simultaneous determination of arsenic, antimony and selenium in environmental soils by hydride generation and inductively coupled plasma optical emission spectrometry

*J. D. Hwang, E. J. Ashford, J. P. Diomiguardi, H. P. Huxley and W. J. Vaughn (Occidental Chemical Corporation, Technology Center, 2801 Long Road, Grand Island, New York 14072, USA)*

The hydride generation technique is unambiguously superior to the direct solution nebulization method in many aspects, such as the improvement of the detection limits for determining hydride-forming metalloids. However, one problem inherent to hydride generation, that has delayed successful application of this method to the commercial ICPs, is that the significant amount of gaseous by-products of hydride reaction ($H_2$, $H_2O$ and $CO_2$) can extinguish a medium or low power ICP source when using the continuous flow mode hydride generation approach.

In this study, a simple and very inexpensive continuous flow *in situ* nebulizer-hydride generation system with an ICP-AES was used for the determination of arsenic, antimony and selenium in environmental samples. The application of hydride generation produces a separation and a higher transport efficiency of arsenic from the sample matrix. Thus the spectral interference and sensitivity problems encountered using conventional pneumatic nebulization are eliminated. The acid content needed for this hydride generation method is the same as the acid content used in conventional nebulization. Therefore, a sample used for simultaneous multi-element determination using conventional nebulization ICP can also be used for hydride generation ICP. The key features of this method which distinguish it from previous works in this field are: (1) it greatly reduces the amount of time needed for sample preparation; (2) only a minimal and inexpensive modification of existing standard equipment is required; (3) only a low/medium power plasma is required, in contrast to the high power plasma required with other methods.

This method has instrument detection limits of 1·0 ng/ml for the As 193·759 nm line, 1·8 ng/ml for the Sb 206·833 nm line and 1·2 ng/ml for the Se 96·026 nm line, respectively. The linear calibration range is over three orders of magnitude, starting from the detection limit.

### A new technique for automatically interpreting ICP-MS spectra

*D. Polk and J. Zarycky (SCIEX, 55 Glen Cameron Road, Thornhill, Ontario, Canada, L3T 1P2)*

*R. Ediger (Perkin-Elmer Corp., 50 Danbury Road, Wilton, Connecticut 06897, USA)*

The Inductively Coupled Plasma-Mass Spectrometry (ICP-MS) technique is ideally suited to automatic procedures for spectral interpretation. ICP-MS spectra obtained in a single, rapid scan (<1 min) contain information for a wide range of elements. Spectral interferences, although present to a limited extent, can be corrected for, permitting detection of virtually all elements of interest (H, O, N, and Ar excepted). To take advantage of the information available, we have developed a new procedure for automatic interpretation of ICP-MS spectra. Previously, solutions have been attempted with numerical approaches such as the least squares technique and linear programming (i.e. simplex) techniques. In contrast, the new procedure uses heuristic, knowledge-driven routines in combination with numerical calculations. This approach is called 'TotalQuant' in recognition of its ability to work with the total information content of the mass spectrum.

The first phase of interpretation involves the assignment of intensities to elements. Rather than perform exhaustive calculations, heuristic or rule-of-thumb routines are used to quickly arrive at suitable results. An example of a heuristic routine can be stated as: 'If an element has been assigned an intensity then the associated oxide intensity will be a fraction (0–10%) of the element's intensity'. There are several rules of this form that act either to constrain or assign element intensities. The invocation of heuristic routines proceeds in stages adjusting the mass spectrum data as assignments are made. The order of assignment for multi-isotopic elements is dynamically varied to achieve a set of assignments that properly correct for isobaric interferences amongst the elements. In some cases, elemental assignments are not made until a series of polyatomic assignments have been made. This delayed assignment of element intensities allows for proper correction of polyatomic interferences.

Following the assignment of element intensities the program performs a semi-quantitative analysis of concentrations for those elements found in the sample. A set of relative response factors for each element is applied to the element intensities to arrive at the corresponding element concentrations. The response factors can be calibrated using an external standard having up to 24 elements of known concentration. Also, samples can have up to 24 internal references. This calibration procedure allows for proper compensation of signal suppression due to matrix effects and for correction of machine drift. There is also a calibration procedure for solid sampling by laser vaporization.

The TotalQuant program can be considered as a combination of artificial intelligence techniques and numerical calculations. The result is an interpretation of mass spectra similar to that produced by human analysis. The program, however, offers the advantage of fast, convenient operation (with calculation times of 1 s). The program is an excellent tool for prescreening samples of unknown composition. Where standards are available, semi-quantitative analyses of concentrations can be performed that are accurate to within 2% for solutions and 5% for solid samples.

### Automation of the USEPA's contract laboratory program requirements for the determination of elements in waste waters by inductively coupled plasma emission spectrometry (ICP-ES) and graphite furnace atomic absorption spectrometry (GFAAS)

*Fred Delles, Douglas Shrader, Alan Marks, Michael Knowles, Tran Nham, Flav Finotello and Brian Allen (Varian Associates, 201 Hansen Court, Wood Dale, Illinois 6091, USA)*

Laboratories entering the Environmental Protection Agency (EPA) Contract Laboratory Program (CLP) are required to follow stringent quality assurance/control procedures to ensure validity and security of analytical data. The number of test solutions which must be regularly analysed restricts the number of sample analyte determinations and requires close attention to detail on the part of the operator.

This paper examines methods for the automation of USEPA CLP requirements for both Inductively Coupled Plasma Emission Spectrometry (ICP-ES) and Graphite Furnace Atomic Absorption Spectrometry (GFAAS) as applied to the protocol requirements for elemental determinations in waste waters. Complete automation of the CLP requirements consists of three phases: testing, actions and reporting.

For ICP-ES, a fully integrated software design produced a command line driven user interface, in a customized multitasking operating system running under MSDOS. CLP test actions are triggered by QC action commands which may be attached to each sample label. The sample label then indicates the file containing the required testing parameters such as true concentrations and upper and lower action limits. A QC Protocol page allows specification of the action to be taken as a result of the text and is used to enter limits for tests which cannot be included in the file structure.

For GFAAS, two designs were required to implement the software, for spectrometers controlled by an IBM compatible PC and for spectrometers controlled by data stations. For the IBM PC, a modular, application software design has been implemented to produce a softkey driven interface under a customized, multitasking operating system under MSDOS. The application module has been added to the spectrometer software to produce a new version which replaces existing spectrometer software on installation. One new interface page controls all of the nine tests in the Quality Control Protocol (QCP). The design for data station controlled spectrometers included consideration of time and date stamping of all results without the convenience of a PC clockcard and the fully automated switch to the method of standard additions on spike failure.

The GFAAS software design triggers CLP test actions in three ways:

(1) At user-defined rates.

(2) On the basis of analytical results.

(3) At pre-defined locations in the sample sequence.

Both ICP-ES and GFAAS designs include an interactive reporting system which flags analytical results and produces error messages in accordance with USEPA requirements. In this paper implementation and application of each CLP test is discussed in detail.

### Design and optimization of a flow injection system for enzymatic determination of sugars

*K. S. Kurtz and S. R. Crouch (Department of Chemistry, Michigan State University, East Lansing, Michigan 48824, USA)*

Immobilized enzyme reactors are used in a flow injection analysis (FIA) system to provide selective, automated determinations of sugars in complex mixtures, such as food samples, without prior separation. The system described can simultaneously determine six nutritionally important sugars: glucose, galactose, sucrose, maltose, lactose, and fructose. Each sugar is reacted enzymatically to produce hydrogen peroxide which subsequently yields a coloured product via a leuco-dye, peroxidase reaction. Separate FIA manifolds containing the appropriate enzyme reactors allow for simultaneous determination of the individual sugars. In optimization of the methodologies, high sensitivity is desired with minimal dispersion. The manifolds are optimized independently, which permits specific conditions to be found for each determination and results in optimal system performance.

The design of each immobilized enzyme reactor is an important consideration in developing the FIA manifolds. Different reactor designs result in different dispersion characteristics due to chemical and physical processes. Packed bed reactors, single bead string reactors, and embedded reactors are characterized and compared for each enzyme. Reactors are evaluated based on their apparent enzyme activity and the dispersion introduced. The optimal reactor design is determined by sensitivity and throughput considerations.

The manifolds are individually optimized for rate-dependent parameters such as pH, flow rate, reactor length, and reagent concentrations. A modified Simplex procedure utilizes a defined response function where system factors are weighted and expressed in a mathematical equation. The response factors considered in optimizing the sugar-determinations are sensitivity, precision, and sample throughput. These factors are measured experimentally and expressed in the response function as the peak height, standard deviation, and appearance time of the peak maximum. The form of the response function optimized varies slightly from one manifold to another to account for other factors such as reagent and enzyme stability.

Optimal system performance is accomplished by combining the results from the reactor design evaluations and the optimal conditions obtained from the Simplex procedure. The results of these studies and their effect on improvement of the sugar determinations over initial conditions are discussed.

### Development of a data acquisition and analysis system for measuring protein binding constants through ultracentrifugation

*Gerald L. Fitzgerald, William J. Cassano, Karl K. Soneson, Frank L. Tobin and Preston Hensley (Smith Kline & French Laboratories, M/S L-331, P.O. Box 1539, King of Prussia, Pennsylvania 19406-0939, USA)*

The ultracentrifuge is an important bioanalytical instrument that has long been used for determining the molecular weight and physical characteristics of proteins in solution. More recently, ultracentrifugation has received attention as a method for quantitatively characterizing protein–protein recognition [1]. This talk will focus on designing a data collection and analysis system for a Beckman Model E Ultracentrifuge (circa 1950). Data collection must be capable of sampling rates in the range of 2 MHz in order to take advantage of rotor speeds as high as 60 000 rpm. The ultracentrifuge's photomultiplier tube has been directly linked to a CAMAC A/D converter, allowing characterization of the reference and sample intensity peaks. The output from the photomultiplier is collected while peak heights are averaged at each radius until the signal-to-noise ratio ratio reaches a predetermined level. Emphasis has been placed on statistical noise control over short and long time periods in order to provide consistent signal to noise ratios at different rotor radii and signal levels.

A second aspect of this work has been to model several properties of this data system including the effects of noise, number of data points collected, and A/D precision. These properties have been evaluated with respect to their effect on the calculations for determining molecular weight and concentration. The goal has been to develop an approach such that the data analysis can compensate for bias from the instrument and data acquisition system.

1. HENSLEY, P. *et al., Journal of Biological Chemistry,* **261** (1986), 11038.

### Guidelines for the selection and integration of computer software and data systems into the small analytical laboratory

*Jeri S. Roth and James A. Kelley (Laboratory of Medicinal Chemistry, Developmental Therapeutics Program, Division of Cancer Treatment, Building 37, Room 5C-02, National Cancer Institute, NIH, Bethesda, Maryland 20892, USA)*

Recently there has been a proliferation of software intended for use in the analytical laboratory on personal computers. These range from simple software packages that perform a specific function such as graphing or statistical analysis to complete systems that collect and analyse data from several instruments, or, in the case of a LAN, from several laboratories. Our laboratory is the analytical component of a medicinal chemistry group involved in the design, synthesis and preclinical development of new antitumor and antiviral agents. Our functions encompass determining the structure, purity and chemical and physical properties of these new compounds, as well as conducting preclinical and clinical pharmacology studies to determine the *in vitro* and *in vivo* disposition, metabolism and pharmacokinetics of potential new drugs. Gas chromatography, high-performance liquid chromatography and mass spectrometry are our major analytical tools.

This paper will discuss our experiences and the guidelines we have developed as a small analytical laboratory for selecting hardware and software packages to carry out the above functions and to automate our instrumentation. In addition, some methods to integrate programs, peripherals and instrumentation and to keep them running smoothly will be outlined. Finally, specific examples will be given of problems we have encountered purchasing and using certain programs and how to avoid these pitfalls.

The first step is to determine what types of packages are necessary. In general, it is best to select the highest level system first so that other programs purchased will be compatible with it. If using a modular approach, try to determine if files or data can be passed between programs. Also, some programs may be standard for a particular company or agency, such as WordPerfect for word-processing. Other factors to consider are cost, hardware and peripheral requirements, and size of the laboratory. If the personnel turnover rate is high, generally the case at a training institution such as NIH, it is more important to choose software that is easy to learn. Also, a modular system with many simpler and self-contained programs may be more useful than one complicated integrated system. A modular approach is often necessary, in any case, to meet all the programming needs of a specific laboratory and to allow for future expansion.

There are definite advantages to purchasing an entire system from the same vendor, especially for the higher end packages. Then, if the LAN or data system does not work, there is one clear responsible party. When buying software for an existing computer, get the hardware requirements in writing before the purchase. If possible, try out a demonstration program first to make sure the software really does what is expected.

As soon as all systems are running, begin to write protocols for correct use. The larger the laboratory, the more critical this is. It is also a good idea to have one designated computer resource person. If your company or the vendor supports certain programs and offers training, take advantage of it. Finally, when adding new software, remember not to overload one computer with too many heavily used programs.

### Computers in the laboratory: chemical information management

*Don Kuehl and Steven C. Simonoff (Galactic Industries Corporation, 395 Main Street, Salem, New Hampshire 03079, USA)*

The multitude of instrumentation in today's analytical laboratory produces a voluminous amount of data. LIMS (Laboratory Information Management System) provides a computerized way to organize work schedules, track samples, and collate results of tests. These systems are mainly text based and usually provide little support for managing and utilizing the raw data. A system which allows processing and archiving of raw instrumentation data could be termed a Chemical Information Management System (CIMS). Such a system would allow easy access of data from a variety of instrumentation on a common computing environment. Due to the multi-vendor approach of most laboratories, simply transferring the data to a central facility is a formidable task. An even more difficult stumbling block to a viable CIMS system is the lack of any standard instrument file format. Nearly every instrument, even those from the same vendor, use different file formats. Furthermore, the CIMS system must provide processing capabilities which are unique to a variety of analytical techniques.

In this paper we will describe a software system under development over the past three years. The system is capable of accepting data from a multitude of instrumentation and provides extensive data processing capabilities. The system is easily networked both locally and through central computing facilities and wide area networks. To date, the system has successfully been used with chromatography, UV/VIS, NIR, MIR, mass spectrometry, X-ray diffraction, Mössbauer, NMR, and fluorescence instrumentation.

### Instrument interfacing: an integral component of laboratory information management

*Richard Earls and Louis Ciabattoni (Perkin-Elmer Nelson Systems, Cupertino, California 95014, USA)*

In many cases, a Laboratory Information Management System (LIMS) is used primarily for sample tracking, work scheduling, manual data entry and report generation. However, another important component of LIMS is the automatic transmission of data directly from the analytical instrument to the LIMS database. Because of the variety of transmission protocols and data formats, it is difficult to provide a universal method of transmitting and receiving data from analytical instruments. This is further compounded by the fact that the transmission of the data from many instruments is asynchronous and 'one shot' in nature. Also, other instruments require bi-directional communication to be most effective.

The implementation of this process can be carried out via a software or a hardware approach. Both approaches include software and hardware components with varying degrees of cost and performance. Several of these approaches of interfacing instruments to a LIMS are examined in this paper. The relative merits of each approach is discussed. Also, the application of these types of interfacing to 'automatic', 'semi-automatic', and manual data entry is described.

### Development of a low-level LIMS-instrument computer software interface system

*Robert J. Wilkes and Robert G. Megargle (Department of Chemistry, Cleveland State University, Cleveland, Ohio 44115, USA)*

Laboratory Information Management Systems (LIMS) have proven to be beneficial to analytical laboratories reeling under the flood of data from increasingly automated equipment. Benefits from LIMS have been substantial in the traditional areas of test ordering, sample tracking, workstation management, results reporting, and the gathering and reporting of laboratory management data [1]. To maximize these benefits, direct interfacing of laboratory instruments to the LIMS computer is essential. However, this aspect of the automated laboratory concept has remained the weakest or least developed part of laboratory automation [2]. Many examples of laboratory instruments hooked directly to a LIMS (either hardwired or through a network) exist in the literature, and there are some examples of instrument setup being accomplished remotely from a LIMS. In this project, both the instrument control software and LIMS were written in-house, and it was possible to integrate the two more completely than is usually possible when the systems are purchased from separate vendors.

This paper describes a software system for multi-level integration of a DEC PDP-11/23 ICP instrument computer and an analytical LIMS running DEC MUMPS on a PDP-11/24. The system provides the ICP instrument user full automation of analytical sample request downloading, sample run set-up, post-run calculation, result review, batch sign-off, and result upload. Access to all other LIMS functions through the regular LIMS menu system is also available through a terminal emulation mode. All batch/sample parameters are downloaded from the LIMS computer and stored locally on the PDP-11/23, decreasing the continuous load on the LIMS computer. In case the LIMS becomes inoperable for periods of time the analyst still has access to all information needed to perform analyses.

When the analyst wants to run a set of LIMS samples, the available in-process batches are placed on-screen in a menu format which displays all pertinent batch-level information, including batch status. The analyst then chooses which batch to run, and a sub-menu comes up that displays all samples in the batch along with sample information. The analyst can select or deselect specific samples to run from this menu or from the following screen which displays the tests requested for each sample. The analysis software is then automatically set up with the appropriate parameters for the samples chosen. The analyst can specify the solution sequence of the run, inserting blanks and standards within the sample number sequence and repeating the sequence where appropriate. After the dilution factors for each sample are entered manually by the analyst, the samples are run and results calculated. At the end of the run, the analyst can view calibration curves, reject data points, and adjust for spectral interferences. The run as a whole can then be accepted or rejected by the analyst. If the run is accepted, the results can be further manipulated by a mass-balance program which is accessible from the result review screen. The final accepted results are stored in a disk file, and the status of the batch is updated.

When all samples in a batch have been run at least once, the analyst may sign off the batch. In a later step, the instrument computer connects to the LIMS, and the analyst can choose to send results for all signed off batches to the LIMS automatically. Thus, the instrument maintains autonomy from the LIMS and only connects when information transfer needs to take place.

1. Megargle, R. G., *Analytical Chemistry,* **61** (1989), 612A.
2. Janney, R. *et al., American Laboratory,* **20** (1988), 34.

### Automated sensitivity enhancement and sample introduction in supercritical fluid chromatography using solventless injection

*K. Cross, J. M. Levy and A. Rosselli (Suprex Corporation, 125 William Pitt Way, Pittsburgh, Pennsylvania 15238, USA)*

The general detection limit in supercritical fluid chromatography for a 1 μl direct injection onto a 1 mm I.D. packed column is approximately 5 ppm with flame ionization detection. For many applications, most notably pharmaceuticals and environmental samples, sensitivity enhancement is required to enable detection. Current sensitivity enhancement is required to enable detection. Current sensitivity enhancement schemes require manual operation, produce broad or no peaks for fast eluting components, and often involve interrupted solvent flow to the analytical column. This is partially due to the disruption of the phase equilibria from the overwhelming presence of excess solvent molecules as opposed to actual solute molecules during the separation process.

An automated, variable volume sensitivity enhancement scheme has been designed to allow large volumes greater than 100 μl of low concentration sample solution to be chromatographed without the interference of broad solvent peaks, loss of fast eluting components, or interrupted solvent flow to the analytical column. The system utilizes an integrated supercritical fluid chromatograph equipped with automatic multi-position switching valves and control software to reduce or eliminate the volume of solvent in the sample solution prior to injection. The technique will be discussed and data presented showing sub ppm analyses, injection reproducibilities, and typical applications. Figure 2 shows the 50 μl

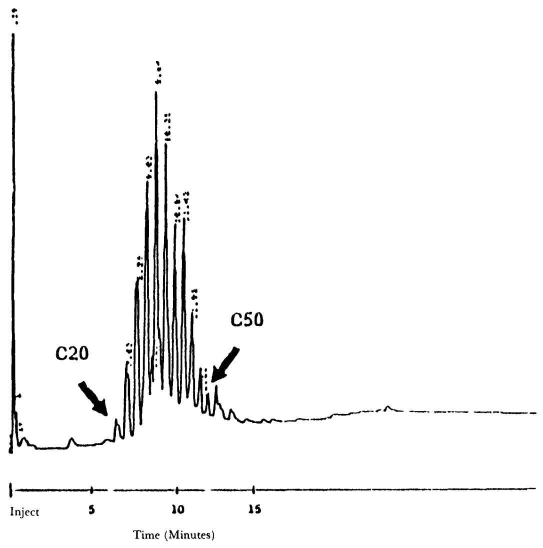

*Solventless SFC injection – 50 μl injection of 1 ppm microcrystalline wax in $CH_2Cl_2$.*

injection of a 1 ppm solution of a microcrystalline was onto a 100 × 1 mm I.D. Deltabond C18 column using methylene chloride as the solvent and flame ionization detection. The results of various parameter optimization experiments for solventless injection will also be presented.

### A rapid sample preconcentration method for trace element determination in animal and plant tissue

*Peter M. Grohse (Research Triangle Institute, Research Triangle Park, North Carolina 27709, USA)*

The effects of environmental contaminants on organisms and their habitats are determined, in part, by the measurement of trace amounts of metals and inorganic compounds in the tissues of fish, wildlife, invertebrates, and waters. In order to provide meaningful background data, it is necessary to employ sensitive analytical methods. Classical tissue and water preparations involve digestions that are lengthy (up to 8 h), prone to contamination at trace levels, and, if utilizing $HClO_4$, potentially hazardous. In addition, multielement measurement techniques, such as inductively coupled plasma emission spectrometry (ICP), rarely provide the required sensitivity for the majority of metallic environmental contaminants. Other measurement methods such as graphite furnace atomic absorption (GFAA), while sensitive, are also time consuming. Consequently, a number of methods such as chelation-extraction and ion exchange have been employed to concentrate a number of elements, although the number of metals concentrated by any one technique are usually limited.

A method utilizing microwave heating that provides enhanced concentrations for over 20 elements is described. The technique is relatively rapid – at least 12 samples are completely prepared in less than 1·5 h – and contamination is minimized. Nitric acid is the only acid employed. ICP detection limits for the resulting digests approach those available from GFAA measurement of 'classical' digests. The technique has been employed on more than 500 animal and plant tissue samples. Elements that have been measured in this manner include Al, Sb, Ba, Be, B, Cd, Co, Cr, Cu, Fe, Pb, Mg, Mn, Mo, Ni, Ag, Sr, Sn, Tl, V and Zn. Spike recoveries are routinely between 90 and 100% (with the exception of Sb, Ag and Sn).

### The use of spreadsheets with analytical instrumentation for laboratory automation

*John M. Graff (National Instruments, 12109 Technology Blvd., Austin, Texas 78727, USA)*

Scientists today are finding that they cannot adequately perform their jobs using manual experimentation methods. The costs, inefficiencies, and potential for errors are simply too high. The low cost and increasingly high performance of personal computers has led many scientists to choose such machines as the foundation for automating their laboratories.

The first step toward automation in many laboratories is the use of a computer to analyse and store experimental data. The methods for performing these tasks range from user-written BASIC programs and general-purpose spreadsheet packages to specialized analysis packages. Although the spreadsheet is best known for its business applications, it can safely be called the most popular analysis software ever written for a PC. A spreadsheet is a natural place to tabulate, analyse, and display data, as many scientists ahve discovered. The problem for many scientists who have used a spreadsheet for analysis and presentation of data is that they could do so only by manually entering the data or storing it in an ASCII file and importing it into a spreadsheet.

This presentation focused on the use of Lotus 1-2-3 and Measure, which works within 1-2-3 to form a comprehensive data acquisition and analysis package that meets a range of general requirements for laboratory automation. Measure adds data acquisition drivers to 1-2-3, allowing data to be acquired from *within* 1-2-3. Measure provides instrumentation control and data collection through the IEEE-488 and RS-232 interfaces. One of these interfaces is found on most analytical instruments equipped for exploring data. For direct data acquisition from sensors such as electrodes, flow meters, and temperature sensors, Measure supports plug-in data acquisition boards.

At the same time that the personal computer has made computing power more accessible, instruments have been designed to be faster and more sensitive. A key design feature is the inclusion of an interface for transferring data direct to a personal computer, such as the RS-232 and IEEE-488 interfaces.

However, the interface on the instrument is only the first step. Most instruments send data as a string of ASCII alphanumeric characters, often sending several pieces of information in a single transmission. As a result, the data received by the computer must be separated or parsed into meaningful groupings, or elements, of data before they are usable. Measure is designed to simplify the complicated task of parsing data into elements such as header information, numbers, words, and status indicators.

Personal computers and instruments create new possibilities in the laboratory. Flexible software environments, such as the spreadsheet, can meet these standards, while providing compatibility with a wide range of instruments by meeting the individual instrument requirements. The goal of the Lotus 1-2-3/Measure combination is to take the information from the instrument and make it immediately useful.

### Design of a PC-based chromatography data system that uses HP integrators

*Jeff Justice (Justice Innovations, Inc., 465 El Capitan Place, Palo Alto, California 94306, USA)*

This paper describes CHROM PERFECT which is shown diagrammatically below:

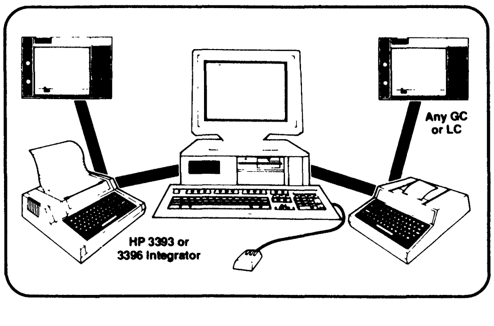

*Diagramatic representation of CHROM PERFECT.*

One or two HP 3396 or HP 3393 integrators digitize the chromatograms and send the data in real time to the PC via RS-232 cables. The PC receives the data and logs it to disk as a background task; thus the PC is available for running other DOS programs during data acquisition. The background task requires only 50 kilobytes of memory, which leaves plenty of memory for other programs to run.

The software contains an advanced peak detection and integration algorithm that performs well with signal-to-noise ratios of less than 5 to 1. As the HP integrator is capable of digitizing to the nearest 0·167 microvolt-second, the system can easily handle peaks less than 1 microvolt high.

The operator interface contains many unique features. Menu selections can be made by mouse, arrow keys, letter key, or special function key. Good default answers are always provided. Limits on entries are displayed with the prompts and checked immediately.

All standard displays from Hercules monochrome to VGA are supported. Mouse-driven graphics allow instant expansion and measurement of the chromatogram. Integration parameters can be quickly modified and updated with a graphic-aided method developer.

The software automatically updates the calibration file and maintains internal or external standard response factors for up to 1000 compounds and up to 60 points of calibration. Calibration fits can be straight line through cubic and weighted equally or by inverse amount.

The *Compare* program allows graphical comparison of multiple chromatograms on screen, printer, or XY plotter. Separate scale factors for each chromatogram compensate for drifts in retention time and amount.

The *Results* software allows tabular comparison of the integrated data from many runs. The data is loaded into a three-dimensional matrix and then viewed like a spreadsheet. Averages and standard deviations are calculated for each row and column. Bar charts and line charts can be quickly plotted.

The *Batch* software allows multiple files from disk to be reprocessed. During reprocessing, new plots and reports are produced.

*Report Write Plus* allows the chemist to customize the report format and calculate results from formulas. *Programmer's Package* gives users documentation and sample source code for reading and writing all Chrom Perfect data files from BASIC and C.

### Main-frame software development for simultaneous multicomponent analysis in near infra-red spectroscopy: application to complex biological suspensions

*Fabrizio Carta, Marco Conti (ENIRICERCHE S.p.A., 00015 Monterotondo, Roma, Italy) and Marco Ferrari (Department of Biomedical Sciences and Technology, University of L'Aquila, 67100 l'Aquila, Italy)*

The recent availability of optic fibers near infra-red spectrometers makes attractive the industrial and biological process monitoring and real time control [1, 2]. In the fermentation process control, in particular, many parameters, such as active biomass, pH, ethanol, glucose, acetic acid and proteins, must be evaluated for the optimization of the overall process and an high degree of interaction is present due to the spectral overlappings in the regions of the water bands. The presently available commercial instruments do not allow for the analysis of a large number of components and the manipulation of a large set of calibration spectra. In addition the calibration accuracy is restricted by the low level of flexibility of the employed mathematical models.

We simulated 110 randomized fermentation conditions with six components varying from 1 to 10 g/l in aqueous solution. Spectra (800–2500 nm) were collected with a Bran+Luebbe InfraAlyzer 500. The best calibration for single component like ethanol was found with 4 wavelengths algorithm (prediction accuracy ±0·75 g/l) but the software was not able to reach an adequate accuracy in the simultaneous determination of all components. To overcome these problems we transferred spectral data to a main-frame and after a suitable data treatment, as filtering and mathematical transformation, a multivariate analysis over all components was performed on the available data making possible the construction of prediction algorithms.

1. Williams and Norris, *Near Infrared Technology in the Agricultural and Food Industries* (Am. Ass. of Cereal Chemistry, St. Paul, 1978).
2. Ferrari *et al., American Journal of Physiology,* **256** (1989), H1493.

### A new computerized analytical system for water in industrial processes or in the environment

*J. L. Cecile, J. Villeneuve, J. Y. Moal, J. L. Pinault, J. P. Jacquin (BRGM, Dept of Analysis, BP 4009, Orleans Cedex 2, France)*

The objective of this work is to provide a continuous analysis of the water in an industrial process or in the environment, involving a mixed solid/liquid medium. The particle-size of the solid material is from a few tenths of a micron to several hundred microns. The problem to be dealt with is therefore that of solid/liquid separation, so that a liquid fraction free of solid particles is always available. The solution obtained in this way can be analysed by any conventional method, but another objective is to obtain the values measured as soon as possible after the solid/liquid separation. Finally, it is useful to have available a system which can analyse at several points in a circuit.

As a solution to the first problem, a non-clogging continuous filtration system has been developed. It is based on the principle of cross-linked filtration and named ARCA. The solution passes through a set of measuring instruments, PIRANA, conductivity, dissolved oxygen, visible UV detectors, or Syrano XRF, as well as sequential measurements when the sample of clear liquid has to be mixed with reagents (Autolab module). The originality of this equipment lies in the fact that it effects the measurements as soon as filtration has been carried out, with no delay for transfer. The MULTIFLUX model enables five points to be checked with a single filtration and analysis tool. The whole system is automated and coupled to a mcirocomputer which controls the different functions and ensures data acquisition and processing using ADOC software.

The results of a programme of tests lasting several months in a semi-industrial unit show the reliability and flexibility of this system. The signals acquired can then be processed. Models of correlations, both between different parameters and between parameters and results of the process, are being developed.

### High temperature thermal desorption autosampler and pyrolysis applications

*D. Grubbs and A. D. Bashall (Carlo Erba Instruments/Fisons, 244 Saddle River Road, Saddle Brook, New Jersey 07662, USA)*

The analysis of catalysts, polymers, rocks and emission by-products (particulates) can be analysed and identified by a high temperature thermal desorption autosampler (HT TDAS) coupled with a gas chromatographic system in a quick and efficient way. The TDAS 5000 system is based on the technique by which volatile materials (organic contaminants) are trapped by adsorption on porous polymers and subsequently displaced by thermal desorption dynamic headspace or pyrolysis and analysed using a gas chromatograph. No special extraction method is needed in this high resolution gas chromatographic system. Designed for unattended operations, the thermal desorption autosampler (TDAS) can make consecutive and reproducible analyses of multiple samples into the injector of the gas chromatograph.

The TDAS unit can be used with capillary and packed columns with a vaporizing sampling system, for example Grob-type split-splitless injector. Other features are high temperature (TDAS) work, programmable (TDAS), pyrolysis, and the TDAS can be configured with a cold trapping option. This cold trapping accessory ensures that the 'sample plug' being transferred to the GC is kept small in order to avoid undesirable band broadening.

The TDAS can be used in many applications, such as the determination of organic pollutants in air and water, direct determination of volatile components in very high boiling matrices and, when needed, the TDAS can be configured for simultaneous multiple detection, for example FID, ECD, SSD and NPD.

The operating parameters, the system design, and data generated by the TDAS unit and results illustrating multiple level desorption and/or pyrolysis of a wide variety of samples will be discussed.

### An evaluation of automated ICP background intensity estimation approaches

*M. L. Salit and J. B. Collins (The Perkin-Elmer Corporation, 761 Main Avenue, Norwalk, Connecticut 06859, USA)*

Two algorithms to automatically select appropriate wavelengths for background intensity estimation in the ICP are presented, evaluated, and compared.

The algorithms described are based on very different approaches towards spectral interpretation: the first approach is based on the heuristic interpretation of the spectral data, while the second approach is based on a statistical interpretation of the spectral data. The heuristic rule based approach to the selection of background estimator wavelengths uses the characteristics of the second derivative to identify candidate wavelengths for background correction, these are then ranked. The 'best' ranking wavelengths are used to interpolate the background intensity at the analyte wavelength. The statistical approach uses an iterative curve fit procedure, masking those wavelengths that are deemed unsuitable for background estimation by their large residual deviations from the fitted background curve. The curve resulting after iteration to mask these outlier wavelengths is interpolated to estimate background at the analyte wavelength.

Both of these approaches are intended to:

(1) Free the skilled analyst from the burden of selecting appropriate background estimation wavelengths for the multiple elements routinely determined in an ICP analysis.

(2) Offer dynamic selection of background estimator wavelengths to ensure that unanticipated interferences do not cause poor estimates of the ICP background intensity at the analytical wavelength.

The nature of the applications performed with ICP-AES demands the benefits afforded with automated spectral interpretation schemes such as those described and evaluated. The large number of samples and sample types calls for reliable automation of as much of the analysis as possible. The demands created by the analysis of samples with complex matrices make it difficult to select fixed background estimator wavelengths when running a 'typical' sample, as it is sometimes difficult to identify such a sample.

The performance of these approaches is qualitatively and quantitatively evaluated with synthetically generated spectra. Qualitative evaluation will consist of the examination of catastrophic failure modes. Quantitative evaluation will be based on the analysis of bias and variance.

### Simultaneous determination of arsenic, selenium and transition metals by ICAP/AES with on-line ion exchange sample pretreatment

*J. M. Riviello (Dionex Corporation, 1228 Titan Way, Sunnyvale, California 94086), R. M. Manabe (Thermo Jarrell Ash, Menlo Park, California, USA) and H. M. Kingston (NIST, Gaithersburg, Maryland, USA)*

The determination of highly toxic trace elements in environmental samples constitutes a significant fraction of environmental analysis. In particular, arsenic, selenium, lead and thallium are difficult to determine in the complex matrices common to environmental samples. The required detection limits for these elements range from 2–10 ppb. The most widely used analytical technique for these elements is graphite furnace atomic absorption spectrometry (GFAAS). While detection limits below a part-per-billion can be obtained by GFAAS, this technique is slow and suffers from interferences which are common in environmental samples.

For the majority of trace metals analysed in environmental samples, inductively coupled argon plasma-atomic emission spectroscopy (ICAP-AES) is the technique of choice. Unfortunately, the detection limits for arsenic, selenium, lead and thallium by ICAP-AES are in the 25–50 ppb range. In order to enhance ICAP-AES detection limits, we have investigated on-line ion exchange preconcentration and matrix elimination techniques.

This presentation described the ion exchange chemistries evaluated for on-line preconcentration. The ability to concentrate and separate anionic and cationic species using a single ion exchange column were demonstrated. Using a fully automated system consisting of an autosampler, ion chromatography and a simultaneous ICAP-AES, detection limits in the 0·5 to 1 ppb range are achieved in drinking water matrices for arsenic, selenium, lead and thallium. Detection limits for other trace elements range from 0·1–0·4 ppb and are determined simultaneously with the arsenic, selenium, lead and thallium.

### Meeting data quality objectives and managing information exchange in environmental GC/MS analysis

*C. S. Campbell, M. M. Booth and D. E. Smith (Finnigan MAT, 355 River Oaks Parkway, San Jose, California 95134, USA)*

Laboratories performing environmental analyses face two significant challenges related to data production: (1) how to ensure that data are of acceptable quality; and (2) how to manage the flow of information derived from data. This paper described automated software for checking data quality on the host computer of a GC/MS system and it discussed options for transferring information between the host computer and other computers.

The quality assurance (QA) checks in the US EPA's GC/MS methods are designed to ensure that data meet defined quality objectives. These checks include ion-abundance criteria for mass spectrometer tuning, retention times of internal standards, recovery of surrogate standards, and response factors of target analytes. In many laboratories, these items are manually inspected in a data review step performed after the data have been acquired. By automating these QA checks and performing them on the host computer immediately after data acquisition, problems can be promptly detected and corrective action taken.

The large volume of data produced and the large amount of processing required place a heavy demand on the GC/MS system's host computer. By transferring quantitation reports and QA data to another computer, the host computer can be dedicated to data acquisition and automated QA checks. Practical issues of sharing information between Data General, Digital Equipment, Hewlett-Packard, IBM, and PC-compatible computers will be described. A cost/benefit analysis of networking via media exchange, serial port transfer and Ethernet local area networks will be discussed.

### Software and hardware validation in a fully automated sampling system

*Robert W. Giuffre (Hewlett-Packard Company, W 120 Century Road, Paramus, New Jersey 07653, USA), S. Shah and Phil Shuler (Ciba Geigy Corp., 100 Old Mill Road, Suffern, New York 10901, USA)*

In recent years, manufacturers have 'opened' their software oeprating systems in order that users might easily customize them to their specific needs. This openness has led to concerns regarding unauthorized changes in programming and validity of results.

The paper presented will discuss programs written on a Hewlett Packard Chemstation. The equipment used is an HP8452A diode, array spectrophotometer. The spectrophotometer is interfaced to an HP9000 computer Model 310 equipped with 1 MByte of memory and a 20 Mbyte hard disk drive. The reports and spectra are printed on an HP Thinkjet printer. Samples are obtained from a 100 position autosampler. The samples are moved through a flow cell with a peristaltic pump.

Programs have been written on this system in a MACRO progamming language that controls the sampler and the spectrophotometer. These programs fully automate standard and sample input, calibration curve calculations, and complete report generation. In addition, validation programs have been written that process previously stored files of known concentration. The same algorithms used to process the stored files also process the 'real-time' files thus ensuring that the software has not been altered.

The entire system has been made turn-key, thus reducing operator input errors. The operator first chooses the application to run. The software sets up the instrumentation and sampler and through the use of prompts the user inputs standard weights and sample amounts. After all the samples are analysed, calculations and reports are done automatically.

This paper presented the steps taken on both the vendor's and customer's side that ensured GLP was met at all times. In addition, it showed how the Chemstation's open system was made secure from unauthorized changes by removing various sections of the operating code.

### Laboratory quality control with a LIMS

*Peter J. Mulligan, Siamak Rowshan, James C. Wallace, Dianne S. Therry and Earl M. Hansen (Roy F. Weston, Inc., 208 Welsh Pool Road, Lionville, Pennsylvania 19355, USA)*

Environmental testing laboratories have an increasing need for quality control (QC). Many state and federal agencies have extensive QC programs to validate analytical results. Laboratories must analyse more control samples and then chart this data to monitor trends in method accuracy and precision.

Increased sample load has made the storage and charting of QC data extremely difficult. PC based systems such as LOTUS have been used but are limited in size and power. Laboratories need a QC system capable of storing vast amounts of data and able to quickly produce QC charts.

A Laboratory Information Management System (LIMS) has been developed at Weston for environmental quality control charting. The FORTRAN customized LIMS operates on a Perkin-Elmer super-mini computer which is interfaced to a variety of analytical instruments. LIMS tracks all client, sample and analysis information.

QC data is entered into LIMS along with client sample data. LIMS has a unique sample numbering system that ties QC samples to client samples.

All QC data in the LIMS database is entered into a special QC database. This database is defined and indexed especially for QC charting. Each week QC data is automatically copied over to the QC database to keep it up to date. This allows archival/retrieval of QC data to occur independently from the LIMS database.

Calculations are performed on the QC data to determine outliers and control limits. The user can specify calculation date range and override outlier selections. Control limits and other statistical results are stored in a QC method database.

A menu system gives the user many options for quickly producing QC charts. Charts can be generated for a particular analyst, instrument, client, or matrix. The user has the option to produce one or over 800 charts at one time.

Several custom reports were created to summarize QC data. These reports make it easy to review method performance. With accurate information of a problem at hand, decisions can be made to improve analytical performance.

The LIMS system has greatly improved the laboratory's ability to track and chart QC data. This has provided laboratory management with an effective tool for measuring and improving the quality of analytical data.

### Fully automated basket dissolution testing of microencapsulated dosage forms

*John A Steichen (Adria Laboratories, Quality Control Lab, 582 W. Goodale Blvd., Columbus, Ohio 43216, USA)*

Recent developments in robotics have been applied to fully automate tablet/capsule dissolution testing. Dissolution testing is one of the most common, labour-intensive procedures in the pharmaceutical laboratory. The Food and Drug Administration, as per USP

requirements, requires dissolution profiles to be determined on numerous oral dosage forms.

Fully automated dissolution testing generally requires the use of robotics to completely integrate all the sample preparation steps with analysis and reporting. The system described in this paper automatically fills the vessels, tests media temperature, places samples in the vessels, analyses the samples, washes the vessels, reports sampling times, repeats the process for the required number of samples and shuts itself down. All the steps rigidly conform to the United States Pharmacopeia (USP) guidelines.

Furthermore, this system fully automates the USP basket method of dissolution testing. Performance data will be presented to demonstrate precision and accuracy for such critical factors as media volume delivery, temperature control, vessel and sampling carry over and sampling dilutions.

In addition, the system can be used to change different types of media. Parameters such as paddle/basket speed, basket exchange and sampling times are controlled by the system. Methods can be stored on disks for use in repeating an analysis or transferring methodology to another laboratory.

Data profiles will be presented for a specialized extended release microencapsulated potassium chloride product showing percent dissolved versus specified time periods. A novel way for testing the tiny encapsulated potassium chloride beads in the USP baskets will be discussed (patent applied for). Comparisons of manual versus robotic procedures will be discussed to show quality of results and validation. Also, increased productivity will be shown by presenting sample through-put data for a variety of dissolution assays.

### Automated dissolution testing for high performance liquid chromatography

*Robert W. Giuffre and Steve Titmas (Hewlett-Packard Company, W 120 Century Road, Paramus, New Jersey 07653, USA) and Hans-Jurgen P. Sievert (Hewlett-Packard Company, Avondale Division, Rt. 41 & Starr Road, Avondale, Pennsylvania 19311, USA)*

With the increasing use of timed-release drug formulations and the need for determining dissolution profiles of multiple active ingredients, the pharmaceutical industry is starting to turn to HPLC as the preferred technique for dissolution testing. Compared to the conventional UV assay, this adds a new level of complexity to the testing procedure and makes automation an essential design criterion for any HPLC-based dissolution system. A system for automated dissolution testing based on a standard Hewlett-Packard 1090M HPLC System with ChemStation controller was presented.

Software routines on the ChemStation are available to establish user-selectable dissolution parameters defining the methodology, the time course of dissolution and the desired output options. Dissolution samples are collected either manually or automatically from a dissolution bath and transferred by hand to the HPLC's autosampler as they become available. The dissolution samples are then analysed on the ChemStation in a fully automated fashion and appropriate dissolution profiles and reports are generated.

The availability of a Diode Array Detector (DAD) with the 1090M offers an additional capability not usually found in traditional dissolution systems: each peak generated by the chromatographic separation is evaluated with respect to its purity by comparing spectra on the leading and trailing edge of the peak. A user-selectable purity threshold allows the system to flag impure peaks thus increasing the reliability of the dissolution data.

### Automated tablet assays and content uniformity using modular robotics

*Kevin A. Tucker, Albertha Paul and Gordon Johnston (Zymark Corporation, Zymark Center, Hopkinton, Massachusetts 01748 and Zymark Ltd, The Genesis Centre, Science Park South, Birchwood, Warrington, Cheshire WA3 7BH, UK)*

Tablet assays and content uniformity analyses of oral dosage forms are time-consuming tests for Quality Control and R&D laboratories in the pharmaceutical industry. Both types of assays have been successfully automated using modular robotics.

Recent developments in laboratory robotics have made it easier to perform content uniformity and tablet assays for multiple products. The system can weigh samples, dispense multiple solvents, dissolve tablets, perform liquid/liquid or solid phase extractions, filtration, serial dilutions, and injection into one or more HPLCs. Increased tablet capacity is obtained by using disposable cups for volumes less than 150 ml and glass containers that are cleaned and reused for larger volumes.

### Total quality issues in medical laboratories of the United Kingdom

*Alan S. McLelland (Institute of Biochemistry, Royal Infirmary, Glasgow, Scotland, UK)*

The primary quality objectives of a clinical laboratory are:

(1) Fast turnround time for tests.

(2) Wide repertoire, constantly under review.

(3) Understandable results.

(4) Accurate results.

A laboratory will also be expected to provide an interpretive service with good clinical liaison; will alert clinicians to unexpected abnormal results; will participate in clinical research and development projects and will encourage use of the laboratory's database for enquiry and hypothesis testing.

Speed of turnround is the most important objective – in Glasgow we currently analyse and report >70% of our high volume core workload in the same half-day in which it arrrives, and can monitor turnround at time points which are recorded for each request. Turnround requires extensive computerization and Local Area Networks with external gateways to allow results to be delivered to our customers. Repertoire relates to turnround, since specimens which must be shipped to a specialist centre take longer to report.

Clinician comprehension of laboratory tests is assisted by form design, interpretive comment by laboratory staff, who may themselves be assisted by expert systems, and telephone discussions augmented, where necessary, by a ward visit.

Quality assurance is not restricted purely to analytical methods – every report leaving the author's laboratory is assessed against the patients' current clinical diagnosis and the trend of previous results with any discrepancies noted and actioned and accuracy of result also extends to correct linkage of current and past reports on a patient. Ultimately, however, the service depends on the motivation and commitment of the staff. Traditionally, in the UK, analysts, R&D staff and laboratory managers are graduate-level and their primary motivation is job-satisfaction rather than high salaries or rapid career progression. Such staff are expected to acquire professional qualitifications and post-graduate academic qualifications where appropriate. These aspects of quality in UK medical laboratories were discussed in detail.

### Flow injection AAS precise automated determinations at the trace level

*Z. Grobenski, T. Guo, G. Schlemmer and W. Schrader (Bodenseewerk Perkin-Elmer GmbH, D-7770 Überlingen, FR Germany)*

The flow injection mode of sample introduction for flame atomic absorption spectrometry has been shown to provide important advantages over conventional sample introduction. For example, due to the constant aerosol flow into the mixing chamber, the flame conditions remain stable and high concentrations of dissolved solids in the sample solution can be tolerated due to low sample consumption and immediate flushing of the nebulizer/burner system after the sample pulse. These advantages translate directly into significantly better detection limits in real samples with high matrix content or in samples dissolved in organic solvents.

Flow injection offers the possibility of rapid on-line preconcentration or matrix separation on micro columns. A preconcentration, elution and measurement cycle can be completed in less than one minute and offers preconcentration factors of up to 50 and consequently detection limits which are superior over conventional flame AAS by more than one order of magnitude.

Hydride generation AAS is a well proven technique to achieve detection limits in the range below 100 ng/l and consequently offers precisions of better than 3% at concentrations of 1 μg/l.

The hydride generation technique combines matrix separation and analyte determination on line and the technique therefore offers high specifity with only minor interferences in complex matrices.

The flow injection mode of sample introduction offers distinct advantages over conventional batch systems: It can be easily automated, the absolute detection limits are a factor of 50 superior, consumption of sample and reagents are lower by a factor of between 10 and 100 and the speed of analysis is a factor of approximately five higher and approaches that of flame analysis.

The most important hydride forming elements (As, Se, Sb, Bi) have been determined in a variety of samples from the environmental, biological and industrial field. Special emphasis was laid on studying the effect of easily reducible metals on the hydride formation. It was found that interferences in complex matrices can be significantly reduced by using the Flow Injection technique.

A Perkin-Elmer FIAS-200 flow injection system has been used in combination with an AS-90 autosampler and a model 2100 spectrometer.

### Preconcentration of trace elements for AAS and ICP

*Prakash Narayanan, G. Csanady, M. R. A. Michaelis, G. Knapp (Department of Analytical Chemistry, Radio- and Microchemistry, Graz University of Technology, A-8010 Austria) and A. Grillo (Questron Corp., PO Box 2387, Princeton, New Jersey 08543, USA)*

Claims of no need of sample preparation in trace analysis with AAS or ICP is still a dream to come true. Matrix separation from analyte or low detection levels of the analyte signals needs sample preparation. Preconcentration using chemically bonded chelating groups are frequently used for analysis of complex samples. They not only separate the matrix from the analyte but also provide high enrichment factors. Problems associated with such methods are the lack of automation, materials with high capacity and fast exchange kinetics and contamination during handling of samples. Therefore an automated device for sample preparation is an answer to the problem.

Trace • Con is a PC-controlled instrument for fully automated preconcentration of trace elements [1]. The easy-to-use software is menu driven and provides high flexibility for method development for on-line and off-line modes [2] of preconcentration. The standard methods and procedures can easily be edited, aided by an on-screen flow-chart of the apparatus. Selection of individual enrichment factors for each sample and automated preconcentration of up to 20 samples, print out of status report on each sample etc. are provided.

EDTrA-Cellulose, a chemically bonded chelating material possessing high capacity and fast exchange kinetics at high flow rates, has been exclusively developed for the Trace ● Con instrument. Toxic heavy metals or environmentally relevant elements, namely Cd, Co, Cu, Ga, Mn, Ni, Pb, V, Th, U, Zn can be selectively enriched in presence of matrix.

In the online mode the Trace ● Con apparatus can be directly coupled to flame AAS or simultaneous ICP-AES equipments. The transient elution profile of the preconcentrated elements are evaluated for peak height and peak area. The detection limits of the atomic spectroscopic techniques shows improvement by one to two orders of magnitude on coupling to Trace ● Con. Currently the system is being adapted for hydride generation and simultaneous determination of volatile hydrides (As, Sb, Se, Sn). These hyphenated methods have been successfully applied to water samples, pharmaceutical samples [2] and standard reference materials [3].

1. Knapp, G., Müller, K., Strunz, M. and Wegscheider, W., *Journal of Analytical Atomic Spectroscopy*, **2** (1987), 611.
2. Prakash, N., Csanády, G., Wegscheider, W. and Knapp, G., *Journal of Analytical Atomic Spectroscopy*, **4** (1989), 347.
3. Prakash, N., Csanády, G., Michaelis, M. and Knapp, G., *Mikrochim. Acta* (1989), in press.

### A new fluorescence detector for mercury

*P. B. Stockwell (P S Analytical Ltd, B4 Chaucer Business Park, Watery Lane, Kemsing, Sevenoaks, Kent TN15 6QY, UK), K. C. Thompson and A. Henson (Yorkshire Water Enterprises Ltd, Sheffield Laboratory, Charlotte Road, Sheffield S2 4EQ, UK) and M. Moses (Questron Corporation, 4046 Quaker Bridge Road, Mercerville, New Jersey 08619, USA)*

The detection of mercury at the parts per trillion level in the environment both in water and the air is becoming increasingly important. A new Fluorescence Detector System has been specifically developed to detect mercury. This has the advantage of a wide linear dynamic range and inherent sensitivity. The system has been developed to monitor mercury in water, rivers, effluents, sewage sludges and soils. A specific interface has been designed which allows the rapid and efficient transfer of mercury into the fluorescence head. To conform to the environmental protection requirements an Absorption Detector Head which fits directly into the fluorescence detector electronics has been designed and is available as a standard part of the PSA Merlin Mercury Analyser. This has the added advantage of extending the range of application to higher mercury levels.

For air analysis the inclusion of a Galahad Adsorber/Desorber system strips out the mercury in air samples and transfers them in an argon stream to the detector. A fully automatic air monitor with absolute calibration will also be decribed. This provides extremely low detection levels in a rapid time scale.

### Continuous monitoring of volatile and non-volatile hydrocarbons in water by an online water monitoring system

*Joseph A. Valade (GE Silicones, 260 Hudson River Road, Waterford, New York 12188, USA)*

An online water monitoring system was developed and implemented to monitor for volatile and non-volatile hydrocarbons in water on a continuous basis. The water monitoring system consists of a self-cleaning filtering unit, a distributive data network unit, and an online water analyser unit, which combines two process gas chromatographs and a sparging unit. The filter unit is a microprocessor-controlled self-cleaning system which uses a stainless-steel 10 micron filtering media. Two sample lines carry the filtered sample into the water analysing unit, one line feeding the sparging unit and the second sample line feeds sample to one of the process gas chromatographs. This chromatograph injects a sample directly from the sample stream to be analysed for non-volatile hydrocarbons. The sparging unit introduces sample into a sparging vessel at a constant temperature, pressure and flow rate. Here an inert carrier gas is bubbled through the sparging vessel to collect volatile hydrocarbons. Each process gas chromatograph has a constant volume injection modules, valveless-type column switching systems with a back flushing feature and flame ionization detectors. Analytical separation for non-volatiles is done with multi-packed columns, and multi-capillary columns are used in analytical separation of the volatile hydrocarbons. A total of 11 volatile hydrocarbons and four non-volatile hydrocarbons are analysed in this manner. Detection limits for most volatile hydrocarbons measured are 3 ppb and measured non-volatile hydrocarbons the detection limits are 175 ppb. Since startup in mid 1988, maintenance has been minimal, with uptime being greater than 96%.

The liquid sampling valve used to inject and vaporize a sample for non-volatile hydrocarbons has seen 18 months of service without having to be rebuilt (seals, stem, rings). As a preventative maintenance item the sample lines going to and away from the liquid sample valve are flushed on a weekly basis. The gas sampling valves (one valve on each sparging system) have seen 20 months of continuous service without repair. Inspection of these valves after 12 months has shown no observed wear or particle build-up. Both sparging systems use dual sparging units. Incorporating dual sparging units into the water monitors has given the ability to clean and service one unit while keeping the second sparging unit in service (no downtime for cleaning glassware).

Calibration, reproducibility and stability of results has not been a problem. Control charting component retention times and response factors for one volatile and one non-volatile hydrocarbon demonstrates stability of ±10% variation of the original data observed.

### Applications of chemometrics to chromatography

*James Duckworth and Don Kuehl (Galactic Industries Corporation, 395 Main Street, Salem, New Hampshire 03079, USA)*

Advances in chemometrics allow chemists to mathematically extract information from data which would be difficult or impossible by other means. In chromatography, much effort goes into optimizing the instrumental parameters to provide adequate resolution to perform quantitative or even qualitative analysis. Classical quantitation in chromatography is accomplished by accurately measuring the area under a peak and comparing it to a standard calibration curve. This necessitates good resolution to obtain accurate quantitative results. Curve fitting techniques, such as the Levenberg–Marquardt [1] method can provide accurate areas of poorly resolved peaks. The technique assumes some model bandshape (for example Gaussian) to be fitted to the unresolved bands. Skewed peaks, such as solvent bands, are not modelled well by simple Gaussian bandshapes and thus accurate quantitation of small peaks contained in solvent tails is difficult. In this paper we will demonstrate the use of curve-fitting techniques using model bands which are skewed to accurately fit the peaks. This allows more accurate quantitation of severely overlapping peaks. It will be shown that this method also provides more accurate peak position information which can be used in qualitative applications to improve selectivity.

1. Marquardt, D. W., *J. Soc. Ind. Appl. Math.*, **11** (1963), 431–441.

### Computer-aided optimization of GC runs

*Dean E. Bautz, John W. Dolan and Lloyd R. Snyder (LC Resources Inc., 3182C Old Tunnel Road, Lafayette, California 94549, USA)*

Although it has been discussed in the literature, the adjustment of selectivity in gas chromatographic (GC) runs via changes in temperature programming rates or isothermal conditions is not widely practised. In practice, changes in these conditions are used primarily to adjust the retention of sample components. Increasing or decreasing resolution ($R_s$) for a given column is done via changing the column plate number by using a longer or shorter column. Changes in selectivity (relative peak spacing) are usually achieved by changing the column type. It is generally not appreciated that less dramatic, but equally significant changes in selectivity can be achieved by changes in isothermal temperatures or in temperature program rates.

This paper discussed the development of GC software (DryLab GC) that enables prediction of GC retention and resolution. The predictions are based on the near-linearity of the log k′ vs 1/T relationship. This software allows accurate predictions of retention and resolution based on two initial temperature progammed runs. Once two experimental runs are made, simulations on a personal computer allow the user to optimize the separation by taking advantage of changes in program rates and isothermal holds. Once satisfactory conditions are found, the separation is confirmed with a third experimental run.

### Automation of precolumn derivatization with PITC for the analysis of amino acids from protein hydrolyzates and the physiological fluids

*Michael Meys, Steven A. Cohen and Thomas L. Tarvin (Waters Chromatography Division of Millipore Corporation, 34 Maple Street, Milford, Massachusetts 01757, USA)*

The Pico•Tag method has been widely employed since 1984 to determine the amino acid content of hydrolyzed proteins and peptides and the free amino acid content of physiologic sample. Introduced originally as a manual method, recent instrumentation innovations have resulted in the development of an automated derivatizer that provides for unattended sample derivatization and injection onto the LC system. The automated derivatizer has been employed for the compositional analysis of protein and peptide hydrolyzates as well as for the determination of amino acid concentrations in biological fluids such as plasma and urine. We have previously reported results for the automated analysis of standards and hydrolyzed samples. This work reports additional results from the analysis of physiologic standards as well as plasma (figure) and urine samples. The results of the automated analysis are equivalent to those obtained using manual derivatization in terms of linearity, accuracy and precision. The primary advantage of the automated procedure over manual derivatization is a dramatic decrease in operator time required and the elimination of measurement, transfer and dilution steps which are removed as potential sources of error.

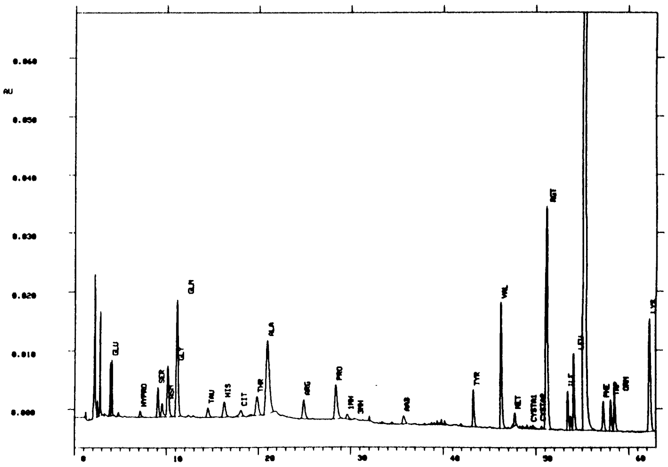

*Plasma analysis.*

### Automated pre-column derivatisation of amino groups of toxicologically and biologically relevant compounds

*M. Steinwand, W. Vogel and Ch. Gönner (Bodenseewerk Perkin-Elmer GmbH, D-7770 Überlingen, FR Germany)*

Pre-column derivatization for HPLC is one of the procedures which demand automated instrumentation and methods. Analysing derivatized compounds enhances selectivity and sensitivity, automation in general results in higher sample throughput and in higher precision and accuracy.

For biologically active amines or amino acids several derivatization techniques have been published. Only a few of them are suited for use in an automated procedure. In this paper, FMOC has been used as a derivatization reagent, employing a new advanced LC Sample Processor for automatic sample handling and injection. HPLC separation has been performed under reversed phase conditions followed by fluorescence detection.

FMOC is known as a fluorescence label which for example allows amino acids in the femtomol range to be detected. A major disadvantage of methods using FMOC as a derivatization reagent is the high fluorescence activity of the reagent itself — which of course is used in excess — and its major hydrolysis product (FMOC-OH). Liquid–liquid extraction can be applied to separate derivatized analytes from FMOC and FMOC-OH before the sample is injected into the HPLC system. As an alternative, in the FMOC-ADAM method 1-aminoadamantane (ADAM) is added to the reaction mixture after the derivatization of the analytes is complete. ADAM reacts with the excess FMOC and builds a highly hydrophobic derivative which appears at the very end of the reversed-phase chromatogram well separated from the compounds of interest. The complete procedure consists of three steps: buffering the sample by adding an appropriate amount of a buffer solution, adding FMOC followed by the addition of ADAM. All these steps are performed using fully automated sample handling and injection. The optimization process of adapting the procedure to the automated instrumentation will be discussed. The results in terms of repeatability, reliability, and time requirements are compared to those from manual operations.

### Fully automated liquid chromatographic analysis of contaminants in food products using on-line dialysis sample preparation

*M. M. L. Aerts (State Institute for Quality Control of Agricultural Products (RIKILT), Bornsesteeg 45, 6708 PD Wageningen, The Netherlands), H. Lingeman and U. A. Th. Brinkman (Department of Analytical Chemistry, Free University, De Boelelaan 1083, 1081 HV Amsterdam, The Netherlands)*

Problems encountered in the determination of contaminants in food products are low analyte concentrations, matrix complexity, and high number of samples. On-line dialysis combined with trace enrichment prior to HPLC, as performed by the ASTED system (Gilson Medical Electronics S.A., France), can overcome these limitations.

The principles, goals, features (compared with classic pretreatment procedures), experimental set-up, parameters influencing recovery/reproducibility and application areas will be outlined. A number of applications solved with this fully automated system will be presented.

As examples, the analysis of a number of drug residues: (i) nitrofurans in eggs, meat, and milk; (ii) sulfonamides in various food products using post-column derivatization to improve the detectability were discussed; and (iii) a fully automated HPLC procedure for the analysis of aflatoxins in milk was shown, using a computer-controlled column-switching system with on-line dialysis, a precolumn containing immobilized anti-aflatoxin monoclonal antibodies, a concentration column, and an analytical column. Finally, the possibilities offered by other selective precolumns such as a silver(I)-thiol precolumn which is used for the determination of the antiviral agent AZT, was overviewed.

Efficiency of on-line dialysis can be as high as 85% in about 3 min using the stopped-flow mode, sample volumes of 100 microliter to over 10 ml can be processed, and depending on the anlytical set-up, 100 samples/day can be automatically analysed.

### Automatic dilution for continuous-flow analysers

*S. C. Coverly (Bran+Luebbe, Werkstraße 4, 2000 Norderstedt, FR Germany), K. Kawamoto and T. Tochimoto (Bran+Luebbe, Sendagaya Shibuya-ku, Tokyo 151, Japan)*

Automatic dilution on a continuous-flow analyser was introduced in 1985 on the Technicon TRAACS system. Since then several alternative methods for obtaining results from samples which fall outside the normal measuring range have been developed for both segmented-flow (SF) and flow injection analysers (FIA). This paper reviewed the current techniques and described a new development.

Two on-line methods are currently used. Sequential flow-cells of different lengths, the shorter being used to measure peaks which produce an off-scale signal in the longer, are applicable to both SF and FIA but restricted to methods with an adequate response above the primary range. It is also possible on FIA systems to measure the peak response at a given point on the decay curve, relying on reproducible timing; in practice, pump pulsations and variations in sample matrix can restrict accuracy.

Methods used up to now which dilute and re-run off-scale samples fall into two categories: those using a special sampler with a mechanical diluter and those which switch from the normal sample flow to a stream of diluted sample. The former is applicable to both SF and FIA but suffers from the disadvantages of cost and complexity while the latter is only applicable to SF methods which satisfy certain hydraulic conditions, and has a limited dilution ratio.

The objective of the described work was to develop a means of automatic dilution to satisfy these requirements:

(1) Applicable to nearly all SF methods.

(2) Equal performance for normal and diluted analyses.

(3) Wider range of dilution ratios.

(4) Extended dynamic range of the system.

(5) Simpler system set-up and operation.

The first method extends the patented valve-switching technique to methods using large diameter sample pump tubes without loss of performance; previously these methods had to run at a lower analysis rate during the dilution run.

The second method uses the valve to switch a dialyser in and out of the analytical stream. As well as allowing a high dilution factor this method allows for the separate, pre-programmed analysis of clean and dirty samples within one run. Both methods use fewer components and are simpler than their predecessor. With the dialyser method it is possible to achieve a ratio of 2500 between the highest and lowest concentrations which can be analysed during one run, compared to 500 previously.

### Flow injection analysis-atomic absorption spectrophotometric determination of ammonia, cyanide and thiosulfate by continuous dissolution of solid silver chloride

*Fatima Esmadi, Maher Khroaf and Abdulrahman S. Attiyat (Department of Chemistry, Yarmouk University, Irbid, Jordan)*

Ammonia, cyanide and thiosulfate have the property of dissolving solid silver chloride. This property has been utilized in their determination, at the microlevel, using the flow injection analysis-atomic absorption spectrophotometric (FIA-AAS) technique. A Tygon tube (1·5 mm i.d.) packed with solid silver chloride was incorporated in a single channel flow system leading to an atomic absorption spectrophotometer. A peristaltic pump was used. Distilled deionized water carrier was pumped through the silver chloride tube to the atomic absorption spectrophotometer. After the baseline was established, known volumes of ammonia, cyanide and thiosulphate standard solutions were injected into the system.

The amount of silver chloride dissolved by these solutions will be proportional to their concentrations. The dissolved silver, transported to the atomic absorption spectrophotometer, produced a signal proportional to its concentration, which, in turn, is proportional to the concentration of the dissolving agent.

The length of the dispersion coil, the length of the silver chloride column, the flow rate and the sample volume were optimized.

A twenty-centimeter dispersion coil was used. Longer dispersion coils were found to reduce the signal. A seven-centimeter silver chloride column was used. Shorter columns resulted in reduced signal, and longer ones gave broader and irreproducible peaks.

A sample volume of 200 μl was found to give a strong and reproducible signal. Higher sample volumes gave irreproducible results. The flow rate was found to affect the FIA-AAS signal. A flow rate of 3·1 ml/min was optimum for the determination of ammonia and thiosulfate. For the determination of cyanide, the optimum flow rate was 3·8 ml/min.

The peak width at the baseline was 30 s. This enabled a frequency of 120 measurements per hour.

The linear working range, the detection limits and the relative precision of each analyte are illustrated in the table below.

| Analyte | Linear working range $\times 10^6$ M | Detection limit (M) $S/N \geq 3$ | RSD (CV) $n = 8$ |
|---|---|---|---|
| $S_2O_3^{-2}$ | 0·5–9 | $1·0 \times 10^{-7}$ | 2.1% |
| $CN^-$ | 0·5–8 | $5·0 \times 10^{-7}$ | 2·4% |
| $NH_3$ | 5·0–100 | $5·0 \times 10^{-6}$ | 1·4% |

### Integrated networking in the Unix environment

*Neerja Raman, Dan Pearce, Tony Beardsley and Doug Durham (Hewlett-Packard Company, Scientific Instruments Division, Palo Alto, California 94304, USA)*

As data systems proliferate and become a more common feature in the Analytical Laboratory, sharing information and peripherals can improve productivity, while decreasing cost. Ideally, one would want to do this in a manner that is straightforward and simple, requiring minimal system management, yet secure and easy to configure. In addition, using industry standards would permit networking between heterogenous systems.

We have developed, as part of the HP-UX ChemStation software, a user interface that meets these goals for ChemStations connected via a local area network (LAN). A user at one ChemStation can perform library searches and process data from files which reside on remote ChemStations. One may also acquire data on a remote system while processing data on the local system. The networking part of the software provides the ability to share files without copying the files on all the stations. In addition, one may share various peripherals, such as line printers and plotters, attached to remote systems.

The HP-UX ChemStation is a good citizen in a multi-OS environment. This means that most of the network transparency that is available between multiple HP-UX systems is also present between DOS and Pascal ChemStations. We use NFS (Network File System – an industry standard) to implement file sharing. NFS operates on heterogenous nodes and a variety of operating systems (OS). Since access techniques are transparent, remote file access is identical to local file access. All of this is a part of the HP-UX ChemStation software. The networking user interface is an extension of the user interface in the data acquisition and data processing part of the software and thus intuitive and consistent.

Details, including performance measures, user interface considerations and benefits were presented.

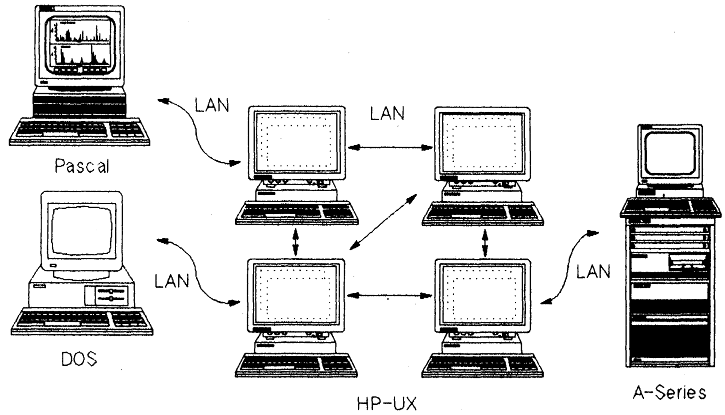

*Diagramatic representation of integrated networking.*

### Automated isolation of DNA from unfractionated (whole) blood

*R. Cathcart, H. Fiske, M. Hane and B. Hoff (Applied Biosystems, Inc., Foster City, California, USA)*

An automated protocol has been developed for DNA isolation from whole blood using Applied Biosystems' Model 340A Nucleic Acid Extractor. It gives consistently high purity DNA ($A_{260/280}$, 1·8–2·0; $A_{230/260}$, 0·4–0·5), as well as high yields, with average sizes >100 kb. Automation minimizes exposure to infectious samples and to toxic chemicals, and also reduces the chances of sample mixups. Up to eight samples can be run simultaneously on the instrument.

0·5–6·0 ml of whole blood is manually injected into glass vessels containing hot Lysis Buffer. The instrument delivers Proteinase K, and the sample is digested for 30 min at 60 °C with gentle rocking. Sample lysates are efficiently extracted twice with 70% Phenol/Water/Chloroform reagent and the phases are separated at 60 °C. A conductivity cell monitors the flow of the lower organic phase to waste. The aqueous phase containing the DNA is retained within the vessels. A chloroform extraction step removes any residual phenol. After sodium acetate addition, isopropanol is delivered to precipitate the DNA. The product is collected on Teflon$^{TM}$ filters within Precipitette$^{TM}$ cartridges, washed extensively with ethanol, and resuspended in a suitable buffer.

The DNA is completely cleavable with restriction enzymes, free of detectable base modifications (determined by HPLC), and active in PCR. Typical yields range from 20 to 40 μg DNA per original ml of blood from a healthy donor. A protocol is currently for use with smaller quantities of whole blood.

### Automatic on-line air monitoring with enrichment down to the ppb-level

*U. K. Goekeler (ES Industries, Voorhees, New Jersey 08043, USA)*

Due to the accumulation of pollution in air and toxicity recognition of chemical compounds and molecules, as well as the increase of regulations, permanent monitoring of the environment and the close vicinity of working areas is highly important. Concentration levels down to the low ppb level should be determined due to the effects of prolonged exposure time for low-level toxic compounds.

Currently, typical procedures involve air bag sampling with analysis in a laboratory, or direct injection of the sample into a GC system. However, these systems are normally either lacking in response time, sensitivity, or stability.

An automatic on-line measuring system, based on enrichment sampling and gas chromatographic separation and detection will be described according to the following:

The sample is sucked through a short, cooled adsorption column for a period of 1 to 5 min. Whereas the matrix (for example air) flows straight through, the higher boiling compounds are retarded. To flush the retarded compounds onto the column, the adsorption column is heated up very rapidly. Capillary columns with column switching are used to separate the target compounds, interference free, and ensure a slender, tall peak. During separation, sample from the next sampling point is trapped. Normally a stable FID is used for the detection.

Advantages of such a system include a ppb or ppt sensitivity level with an enrichment factor of up to 100, interference free detection by capillary column separation, automatic around-the-clock monitoring with an on-line analysis time of 2 to 5 min per stream for up to 15 sample streams, maintenance free cyrogenic cooling, and weeks without recalibration for stable results.

The design and principle function of this system were explained in detail, technical parameters discussed, and results were shown in regard of practical applications done.

### Automatic on-line monitoring of volatiles in water. Prevention of water pollution

*U. K. Goekeler (ES Industries, Voorhees, New Jersey 08043, USA)*

The use of water for process cooling, frequently causes volatile polluting because leaks occur within the plant. The amount of volatiles discharged into the environment or into the biological treatment facility can be significant considering the amount of water used. Manual sampling and analysing of the effluent water, even at frequent intervals, shows possible pollutant emission hours too late; therefore, hindering corrective action to the point that permit violations to easily occur.

An analysing system running continuously on-line, show possible pollutants very quickly at the ppb level. The compounds monitored are organic volatiles (boiling point range up to approximately 150 °C) like aromatic hydrocarbons or chlorinated hydrocarbons.

This is done by using a continuous sparging technique and a Process Gas Chromatograph equipped with capillary columns. Typical analysis times are in the 12 to 15 min range and the sensitivities reached are typically in the 1 to 5 ppb range for individual compounds.

An analysing system with these abilities was introduced two years ago and has been applied numerous times since. In this paper, the applications used for:

(1) Plant water effluent (dirty water);
(2) Water treatment plant effluent (clean water);
(3) Compounds determined; and
(4) On-line water pretreatment before analysis are described.

A significant amount of experience was gained in regard of the applications possible, the required sample filtering, the instruments' stability, the maintenance required, and the benefits gained; all of which were reported.

### Automated solid phase extraction and HPLC injection of theophylline from serum

*Brian D. Holden, Brian G. Lightbody and Julie Tomlinson (Zymark Corporation, Zymark Center, Hopkinton, Massachusetts 01748, USA)*

An automated procedure has been developed for the solid phase extraction and HPLC analysis of theophylline, its metabolites and other commonly occurring xanthines from serum. The automated extraction procedure is performed using a BenchMate Workstation Model B220.

An aliquot is automatically transferred from a sample tube to a process tube where an internal standard and diluent are added to the aliquot and the sample is mixed. The workstation then conditions the solid phase extraction column, loads the sample, washes the column and elutes the component of interest. The collected fraction is automatically injected into the HPLC. Data were presented which showed system performance.

The BenchMate Workstation also offers a unique time-saving feature for an analyst. The validation of each step required for this extraction was done automatically through use of the integral four-place balance. data were presented which demonstrated the reproducibility of liquid handling operations.

### A new sample injection technique for total organic carbon (TOC) analyser

*Nghia Ton, Yoshi Takahashi and Kent Lines (Rosemount Analytical/Dohrmann Division 3240 Scott Blvd., Santa Clara, California 95054, USA)*

Total organic carbon (TOC) is a widely used indicator for organic contaminant levels in high purity water, drinking water, seawater and wastewater. However, laboratory analysers generally lack particulate-handling capability, especially when equipped with an autosampler. As a result, only the dissolved portion of the organics (DOC), rather than TOC, is commonly measured for much wastewater containing various amounts and sizes of particulates.

This report describes a newly developed autosampler which can reliably handle particulate-containing wastewater samples.

The autosampler consists of a sample vial holder (sample tray) and a sampling device. A long inert tubing is connected to a gas-tight syringe through a three-way valve. The third port of the valve is connected to a gas supply. The inert tubing constitutes of a sample probe and a sample retainer.

Prior to taking an aliquot of the sample from a sample vial, the sample probe is immersed into the sample vial and the sample is stirred to suspend particulates uniformly by passing gas through the sampling probe. Then the syringe plunger is extracted to suck the desired amount of the sample into the inert tubing. The valve and syringe do not have contact with the sample. The sampling probe is moved to the injection port of the TOC analyser, and the sample is injected by the gas.

It was found that sample volume between 10 μl and 400 μl could be reliably sampled with very little cross-contamination.

The performance characteristics of the autosampler were reported, as well as the TOC results obtained with various wastewater samples by two different injection devices: the new autosampler and a conventional syringe injector.

### Automatic sampling system for high temperature capillary GC ensuring discrimination free injections over the full boiling point range (0 to +800 °C)

*A. D. Bashall (Carlo Erba Instruments/Fisons, 244 Saddle River Road, Saddle Brook, New Jersey 07662, USA), F. Munari and S. Trestiani (Carlo Erba Instruments, 20090 Rodano, Italy)*

High temperature capillary gas chromatography (HT HRGC) is suitable for thermostable compounds with relatively high molecular weights. The GC hardware, including the capillary column, is today able to achieve analytical separations at maximum oven temperatures in excess of 400 °C. When quantitative results are required, the main problem still remains today the full transfer of the sample inside the capillary column. The cold splitless programmed temperature vaporizing injection technique shows increased discrimination over C50 even if the operating conditions are optimized for enhancing the sample transfer into the capillary column. The cold split injection technique should, in principle, assure better results compared with splitless. It is, however, of limited interest for HT HRGC due to the low solubility of heavy thermostable components. It is very difficult to obtain and manipulate the concentrated solutions needed for split injections.

However, the cold on-column injector is the only sampling technique to show no discrimination, permitting sample injection into a capillary GC system for compounds having boiling points in excess of 500 °C (cf. C50 BP = 575 °C).

### A new method for the automatic and selective determination of total organic carbon in soils, sediments and rocks

*B. Lavettre (Carlo Erba Instruments/Fisons, 244 Saddle River Road, Saddle Brook, New Jersey 07662, USA), M. Baccanti and B. Colombo (Carlo Erba Instruments, 20090 Rodano, Milan, Italy)*

The total organic carbon determination in solid samples is of primary importance in analytical chemistry applied to agronomy, geology, environmental science and in the quality control of some industrial products.

The classic analytical method used for this type of determination, the Walkeley-Black method, is not satisfactory. Its drawbacks are the high cost, the environmental problems due to the use of chromium, and it is labour intensive.

Two alternative methods have been developed in the past, both based on automatic elemental analysis. The first consists of the pretreatment of the sample by acidification, filtration and drying before instrumental analysis. Acidification ensures the complete removal of the carbonates, but the experimental data does not take into consideration those organic compounds which are soluble in the acid liquid phase and are washed out. Moreover, the calcium salts yielded after the acid treatment are very hygroscopic. The second proposed method is based on the preliminary assumption that all the most common carbonates are not decomposed at 600 °C; so, the elemental analysis performed at low temperature should yield $CO_2$ from the organic matrix, but not from carbonates. Further investigation has shown matrix-effect problems.

In this presentation, the authors will introduce a new, automatic method to definitively solve this analytical problem.

Instrumental elemental analysis is performed at 1000 °C for the organic carbon determination of the sample weighed in a metal capsule, acidified in the same capsule and dried at 80–100 °C. The total run time of the analytical cycle for a single determination is 3 min.

### A new concept of automatic sample handling and preparation for steel plant laboratories

*G. Hawickhorst (HERZOG Maschinenfabrik GmbH, PO Box 2329, D-4500 Osnabrück, FR Germany)*

During the last years different approaches have been made to automate the handling and preparation of iron, steel and slag samples for optical emission, X-ray fluorescence and combustion analysis.

Various types and shapes of samples made it difficult to process those samples under routine conditions with the required reliability.

A certain amount of sample standardization has taken place during the last years so that also a standardization of the preparation equipment required was possible.

New integrated systems are now available which can handle and prepare the samples without any manual interference. So human influence and errors are eliminated and a better reproducibility can be obtained.

Layouts of recent installations in the steel industry and the equipment involved were shown. A new generation of sample handling and preparation machines offers the possibility of fast processing of samples which subsequently leads to remarkable cost reductions in the steel making process.

### Design and performance of an automatic static headspace analyser

*Robert G. Westendorf and Herbert J. Lehan (Tekmar Company, PO Box 371856, Cincinnati, Ohio 45222-1856, USA)*

Static headspace sampling is a commonly used technique for preparation of samples for analysis by gas chromatography. It allows volatile compounds to be removed from nonvolatile matrices that are not amenable to GC injection. Samples are enclosed in septum-sealed vials and thermostated at a selected temperature. Volatile compounds will migrate into the vapour phase, and in many cases an equilibrium will be established between the vapour and sample (solid or liquid) phases. An aliquot of the vapour phase is then removed and injected into the GC for analysis.

An automated analyser has been designed to maximize performance in the most critical areas, while still retaining ease of use. The unit is pneumatically based on the 'valve and loop' principle. Vapour aliquots are removed by inserting a needle into the vial and pressurizing it. This pressure is then allowed to vent through a valve and fill a sampling loop. The valve is then rotated to allow the loop to be backflushed with carrier gas to sweep the sample into the column.

Samples are heated over a range of 40 to 200 °C in a twelve-position, electrically heated block. Additional samples can be added or removed automatically via a 50-position autosampler. Use of the autosampler allows the feature of constant heating time to be used, ensuring that each sample is heated for a precisely reproducible time before sampling. A vial agitator is included for mixing of samples to reduce the equilibration time.

The unit is controlled through a tactile-response membrane keypad with an interactive LCD display. Up to four methods can be run automatically. Complete automation is provided for operation with any automated GC. Communication with data systems is provided through both BCD and RS 232C interfaces.

### Coupling FIA with an ion-selective electrode for an on-line determination of trace levels of chloride in 20% caustic

*Kevin Hool (Dow Chemical Co., Central Research-Engineering Laboratory, Midland, Michigan 48674, USA) and E. D. Yalvac (Dow Chemical Co., Analytical Sciences, Midland, Michigan 48674, USA)*

Rapid on-line analysis of trace levels of analyte in extremely difficult matrices is a problem frequently confronting an industrial analytical chemist. A method for the on-line determiantion of trace levels of chloride ion in 20% caustic to be used for process control is a problem recently encountered. The need for a dedicated on-line method in a continuous operation mode providing rapid information for feedback process control precludes many proven laboratory instrumental techniques for chloride analysis. Flow injection analysis (FIA) is an analytical technique which meets these criteria and represents an ideal tool for rapid analysis (amenable to control applications) requiring minimal cost.

Flow injection procedures for the determination of chloride have been based on displacement of thiocyanate ion from its mercury complex by chloride [1–3]. The thiocyanate forms coloured complexes with iron (III) so that chloride can be monitored spectrophotometrically. However, this method is not ideally suited for the on-line determination of chloride in caustic because of the toxic reagents used and due to the formation of ferric hydroxide precipitate within the Teflon valve components. The precipitation leads to premature failure of a valve that already has a low duty cycle (i.e. 10 K cycles). This maintenance along with the necessity of using toxic reagents that pose a waste hazard prompted an alternate method of analysis.

We have devised an FIA method incorporating a chloride ion-selective electrode for the on-line determination of parts-per-million (ppm) levels of chloride in 20% caustic. Several FIA configurations have been evaluated in terms of sensitivity, dynamic range, caustic neturalization, and reagent consumption. The evaluation of various materials of valve component construction to improve operation lifetime will also be presented. The FIA schemes presented can be utilized with other ISEs for selected ion analysis.

(1) Ružička, J., Stewart, W. B. and Zagatto, E. A., *Analytica Chimica Acta,* **81** (1976), 387.

(2) Hansen, E. H. and Ružička, J., *Analytica Chimica Acta,* **87** (1976), 353.

(3) Ružička, J., Hansen, E. H., Mosbaek, H. and Krug, F. J., *Analytical Chemistry,* **49** (1977), 1858.

### On-line micro-distillation apparatus for segmented-flow analysers

*S. C. Coverly (Bran+Luebbe, Werkstraße 4, 2000 Norderstedt, FR Germany) and M. Sahn (Bran+Luebbe Analyzing Technologies, 103 Fairview Park Drive, Elmsford, New York 10523-1500, USA)*

Distillation has been applied to segmented-flow (SF) analysers since 1969 for the on-line determination of volatile compounds such as phenols, cyanides and fluorides which must be separated from interfering material before analysis. Some methods also incorporate a full or partial breakdown of complex compounds. More than 100 such systems are estimated to be in current use.

Current SF distillation methods suffer from high dispersion, resulting in a low sampling rate. Due to the higher sampling rates of modern SF systems the distillation becomes the rate-determining step. This is particularly inconvenient on multi-channel systems where the other methods are capable of higher sampling rates.

The objective in designing a miniaturized continuous-flow distillation apparatus was to reduce the disperson within the system to less than one half of current systems without adversely affecting the breakdown of complex compounds.

Experiments showed that a simple reduction in scale of existing distillation apparatus was not effective in reducing dispersion and resulted in unacceptable hydraulic conditions.

The microflow technique uses special glassware combined with modifications of the conditions for distillation and condensation.

The attainable sampling rate is approximately double that of previous techniques. Data will be presented for day-to-day reproducibility and recovery from complex phenol and cyanide compounds and compared to that of previous methods.

